# Innovative Material-Based Wearable Non-Invasive Electrochemical Sweat Sensors towards Biomedical Applications

**DOI:** 10.3390/nano14100857

**Published:** 2024-05-14

**Authors:** Sheng Zhang, Zhaotao He, Wenjie Zhao, Chen Liu, Shulan Zhou, Oresegun Olakunle Ibrahim, Chunge Wang, Qianqian Wang

**Affiliations:** 1 Ningbo Innovation Center, Zhejiang University, Ningbo 315100, China; szhang1984@zju.edu.cn (S.Z.); 22260404@zju.edu.cn (Z.H.); 22125146@zju.deu.cn (W.Z.); liuchen@nit.zju.edu.cn (C.L.); 22360469@zju.edu.cn (S.Z.); 12125126@zju.edu.cn (O.O.I.); 2 State Key Laboratory of Fluid Power and Mechatronic Systems, School of Mechanical Engineering, Zhejiang University, Hangzhou 310027, China; 3 Faculty of Science and Engineering, University of Nottingham Ningbo China, Ningbo 315100, China; 4 School of Biological and Chemical Engineering, Ningbo Tech University, Ningbo 315100, China; 5 Polytechnic Institute, Zhejiang University, Hangzhou 310015, China; 6 School of Mechanical and Energy Engineering, Ningbo Tech University, Ningbo 315100, China; wangchunge@nit.zju.edu.cn

**Keywords:** innovative materials, sweat sensor, non-invasive, wearable, biomedical application

## Abstract

Sweat is an accessible biofluid that provides useful physiological information about the body’s biomolecular state and systemic health. Wearable sensors possess various advantageous features, such as lightweight design, wireless connectivity, and compatibility with human skin, that make them suitable for continuous monitoring. Wearable electrochemical sweat sensors can diagnose diseases and monitor health conditions by detecting biomedical signal changes in sweat. This paper discusses the state-of-the-art research in the field of wearable sweat sensors and the materials used in their construction. It covers biomarkers present in sweat, sensing modalities, techniques for sweat collection, and ways to power these sensors. Innovative materials are categorized into three subcategories: sweat collection, sweat detection, and self-powering. These include substrates for sensor fabrication, analyte detection electrodes, absorbent patches, microfluidic devices, and self-powered devices. This paper concludes by forecasting future research trends and prospects in material-based wearable non-invasive sweat sensors.

## 1. Introduction

Over the past decades, wearable sensors have garnered significant attention due to their versatile applications in the realms of motion recognition, wellness monitoring, disease diagnosis, and digital health monitoring, particularly amid the COVID-19 pandemic [1,2,3,4,5,6,7,8]. After some significant breakthroughs in innovative materials, wearable sensors that promote non-invasive monitoring with relevance spanning a wide array of personalized medical applications have become more popular due to the soft physical construct and thin, lightweight design of these sensing systems [9,10,11,12]. The sensors, electronic circuits, wireless power modules, and communication components are combined together to form ultra-thin, lightweight, stretchable, and low-modulus membranes [13,14]. As a result, wearable sensors exhibit numerous advantageous features, including wireless connectivity, lightweight design, flexible form factor, and compatibility with human skin [15,16].

The rapid development of wearable sensors allows the non-invasive detection of analytes in accessible biofluids, providing a window into the overall dynamic biomolecular state of the human body [17]. Among the body biofluids, such as sweat, blood, urine, tears, saliva, or tissue fluids, sweat stands out owing to its many physiological functions and widely distributed sweat glands, making it an excellent candidate for biomolecular non-invasive continuous monitoring [18]. Wearable electrochemical sweat sensors have emerged as a promising tool for non-invasive monitoring of physiological parameters, providing real-time insights into the biomolecular state of the human body [19]. To obtain comprehensive biochemical information, these wearable devices are designed to detect a diverse range of analytes in sweat, including ions, metabolites, and biomarkers [20]. These sensors employ electrochemical principles to quantify the concentration of specific target molecules, utilizing amperometric, potentiometric, or impedance-based measurements. The widespread distribution of sweat glands across the body facilitates non-invasive sample collection, making these sensors ideal for continuous monitoring [21,22]. Since the first demonstration of wearable sweat lactate sensors in 2013 [23], there has been significant progress in the field, leading to the development of sophisticated devices capable of monitoring a variety of physiological parameters [24]. The potential applications of this technology in biomedicine are vast, holding promise for transformative approaches to patient monitoring and treatment [25].

**Figure 1:**
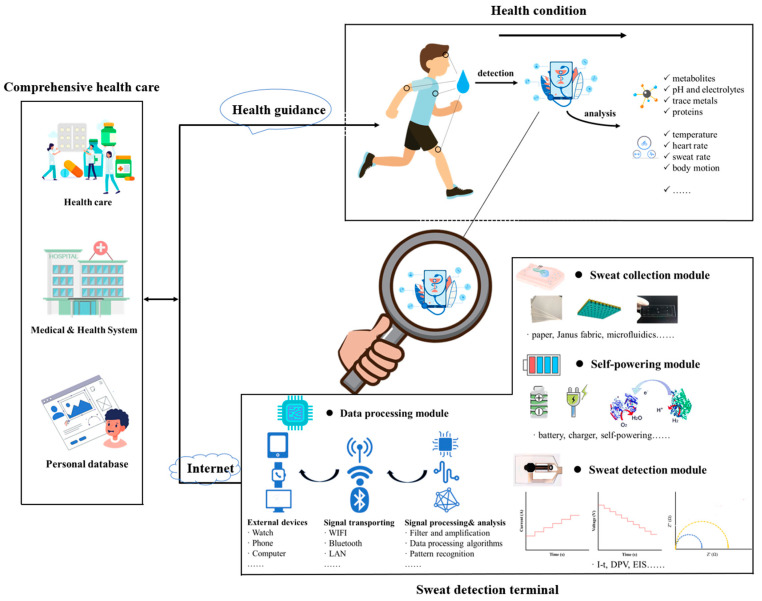
A schematic diagram of a health-monitoring and guidance system based on wearable sweat sensors. The system comprises a sweat sensor analysis terminal and an external comprehensive health care. The portable sweat detection terminal consists of four primary components: a sweat collection module, a sweat detection module, a data-processing module, and a self-powering module. This wearable device is capable of sampling and analyzing sweat, and wirelessly transmitting physical, biochemical, and biomedical information to the user’s terminal, such as smartphones or computers. By establishing a personal health database, the system enables long-term monitoring of an individual’s health status. Medical and health databases contributes to enriching the human health database, thereby enhancing the accuracy of medical health research. Finally, the comprehensive health system provides personalized health guidance, such as diet advice, exercise advice, etc., and feeds back to the individual, aiding in health management.

For the wearable electrochemical sweat sensors, a critical initial step involves the extraction of sweat, which is subsequently directed to a reaction zone for biomarker detection. Additionally, for deployment in real-world settings, it is imperative to incorporate mechanisms for data processing and a stable power supply, as depicted in Figure 1. Over recent years, the research in the domain of wearable electrochemical sweat sensors mainly focuses on three key areas: the creation of sweat collection modules like innovation in sweat adsorption materials and microfluidic architectures, the advancement of sweat detection modules including various catalyst nanomaterials, and the development of self-sustaining power sources through the innovation of new materials. According to the different functions, the typical components of an electrochemical sweat biosensor include the following modules (Figure 1):

- Sweat collection module: Sweat collection modules serve as a pivotal component in wearable healthcare devices, enabling the non-invasive monitoring of physiological markers [26,27]. These modules employ skin patches and microfluidic chips to gather perspiration, which contains vital biochemical indicators of health [28]. The design of sweat collection modules hinges on material selection, microfluidic architecture, and preservation strategies to maintain sample integrity [29]. Advances in material science have led to the use of novel adsorbents and biocompatible substrates, enhancing both comfort and functionality [25]. Concurrently, innovations in microfluidics have improved fluidic control and analytical precision, expanding the scope of applications [30].
- Sweat detection module: Sweat detection modules are integral for quantifying biomarkers in perspiration, utilizing a variety of sensors including biosensing electrodes, nanosensors, and more [31]. These modules consist of a flexible substrate and detection electrodes, with different sensors tailored to detect specific biological molecules or markers [32,33]. Enzymatic sensors rely on enzyme–analyte reactions to generate electrical signals, exemplified by glucose sensors that employ glucose oxidase [34]. Ion-selective electrodes (ISEs) detect particular ions using ionophores with high affinity, such as chloride ISEs for cystic fibrosis diagnostics [35]. Antibody-based sensors bind target molecules, triggering measurable responses, useful for identifying proteins or biomarkers [36]. Nanomaterial-based sensors leverage carbon nanotubes, graphene [37], or metal nanoparticles to amplify sensitivity and selectivity, capitalizing on their unique characteristics and large surface area for efficient analyte detection in sweat [38]. Electrode materials vary depending on the biomarkers under investigation, yet regardless of the detection approach or substance analyzed, the underlying flexible substrate serves as a common platform for different sensing techniques [39,40].
- Data-processing module: The signal-processing module serves as a critical component in the translation and analysis of sensor data, bridging the gap between raw sensor outputs and meaningful biological insights [41]. It commences by transducing signals from physical to electrical formats, potentially employing amplification and filtering techniques to enhance signal quality [42]. Subsequently, the module applies sophisticated analytical methods, including pattern recognition and data-processing algorithms, to extract and elucidate significant biological signatures [43,44]. Furthermore, it facilitates the conveyance of processed data to various external devices and cloud services via a combination of wired interfaces (e.g., USB interface or serial port) and wireless protocols (e.g., Bluetooth, Wi-Fi, RFID, etc.) [45]. Generally, the design of the module integrates proficiency in both electronic hardware and software algorithms, ensuring efficient and reliable data management, which implies that progress in materials science has exerted less influence on the development of this segment [46].
- Self-powering module: The self-powering module offers a sustainable and convenient solution for powering sensors. Unlike traditional power supply units, such as batteries or external adapters, this module harnesses environmental energy, aligning with green energy principles [47]. It is particularly suitable for wearable electrochemical sweat sensors, as it is lightweight, compact, and provides a stable power source without posing any harm to the human body [48]. The self-powering module encompasses various technologies, including solar cells, triboelectric nanogenerators (TENGs), and biofluid cells (BFCs) [49]. These features make it an innovative and efficient choice for sensor applications, promoting both practicality and environmental consciousness. Each of these self-supplied electric methods has its advantages and disadvantages, and it is necessary to choose different technologies for different scenarios, and then choose different innovative materials [50].

The current review extensively covers the array of monitoring techniques and sensor systems tailored for the detection of diverse biomarkers, various detecting electrodes, and varied sensing mechanisms. However, it excludes detailed analysis and discourse on the functional performance of novel materials integrated into sensor devices. This manuscript delves into cutting-edge research advancements in the domain of wearable electrochemical sweat sensors, with a specific focus on the innovative materials employed in their construction. The innovative materials pertinent to the sweat sensor systems are categorized into three subcategories: materials for sweat collection, materials for sweat detection, and materials for self-powering. Furthermore, this paper provides insights into future research trends and potential developments associated with wearable, non-invasive sweat sensors that leverage innovative materials.

This review explores advancements in wearable electrochemical sweat sensors, focusing on novel materials integral to their design. Material classifications include sweat collection, detection, and self-powering. Additionally, insights into future trends and potential developments are also discussed.

## 2. Wearable Non-Invasive Electrochemical Sweat Sensors

Wearable technology has revolutionized the way we monitor and manage our health. One of the most promising areas of wearable technology is in the field of biomedical applications, specifically the development of non-invasive electrochemical sweat sensors. Non-invasive wearable biosensors that monitor sweat have several advantages over sensors that monitor other biofluids (such as blood or urine), including non-invasiveness, real-time monitoring, and a wide range of detectable parameters. These sensors have the potential to transform healthcare by providing real-time, accurate, and continuous monitoring of important biochemical parameters in sweat. The key to the success of these sensors lies in the innovative materials used in their construction, which include innovative materials for sweat collection, innovative materials for sweat detection, and innovative materials for self-powering.

### 2.1. Innovative Materials for Sweat Collection

Sweat collection serves as the initial stage of sweat testing. The expeditious secretion of human sweat can be achieved via two approaches: passive and active methods. In the passive method, individuals stimulate ample sweat production through vigorous physical activity, such as rigorous training. Iontophoresis, on the other hand, represents a commonly employed active technique for sweat induction, facilitating the collection of sweat samples during bodily relaxation [29]. After perspiration occurs, we have various means of collection at our disposal, including the utilization of paper, cotton fabric, fibers, hydrogels, microfluidic chips, etc. [51,52,53].

Wearable microfluidic chips represent an emerging method for sweat collection, wherein they exhibit the capability to effortlessly gather sweat and convey it to areas dedicated to sensing [54,55]. This pivotal function in the realm of wearable devices highlights their significant role. Due to its exceptional chemical stability, high temperature resistance, elasticity, transparency, biocompatibility, and wear resistance, polydimethylsiloxane (PDMS) finds extensive applications in the fabrication of microfluidic channels.

Diverse materials are at one’s disposal for capturing sweat, ranging from paper to sophisticated microfluidic chips, each with unique traits that affect their suitability for various uses [29]. Furthermore, the selection of materials for sweat collection significantly impacts the design and performance of flexible and wearable devices [54]. Each type of material brings unique strengths and weaknesses that can influence the accuracy, reliability, and user experience of these devices. The impact of different materials on flexible and wearable devices can be assessed based on several key parameters, including their mechanical flexibility, electrical conductivity, skin compatibility, and manufacturability [46,56,57,58].

**Figure 2:**
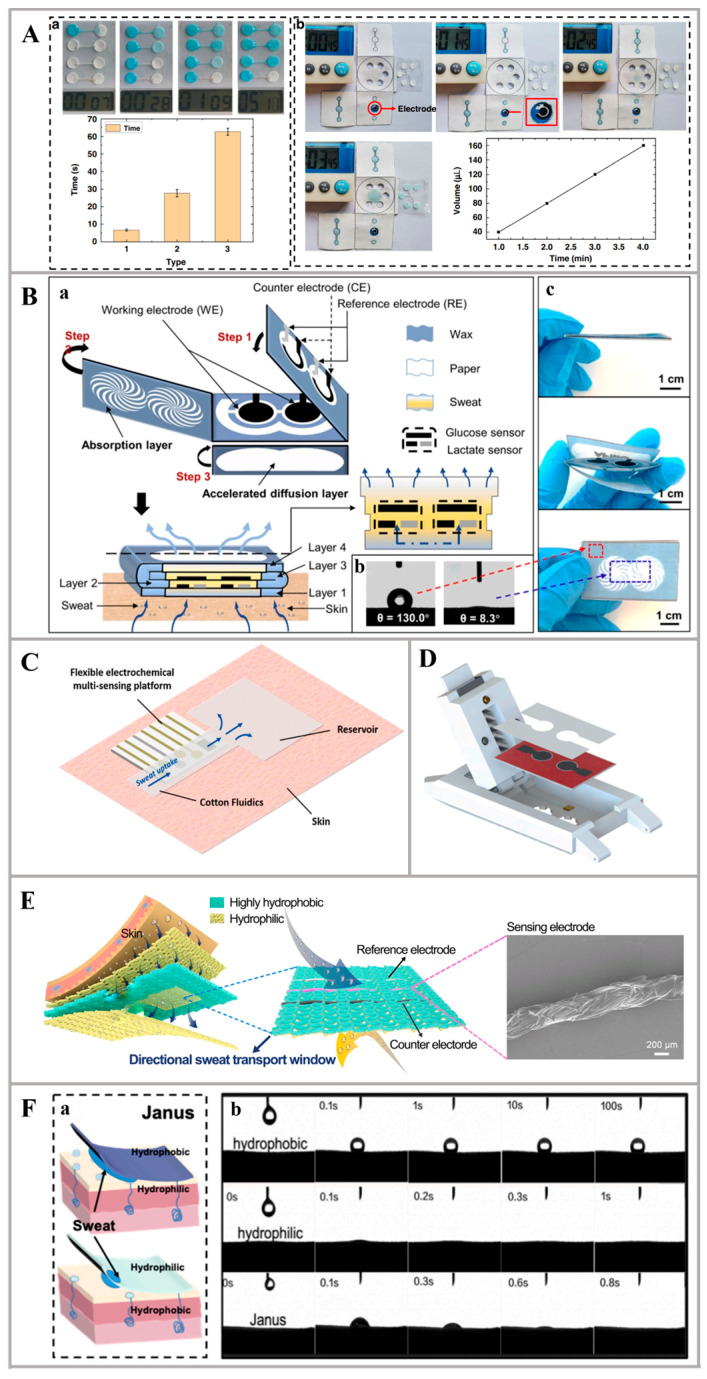
Innovative materials for sweat collection: (**A**) Paper-based microfluidic channels for collecting sweat: (a) Differences in wax immersion times result in variations in the flow times of liquids. (b) Influence of hydrophilic channels [55]. (**B**) Explored view of HIS paper and the tests of hydrophobic and hydrophilic regions: (a) Structural anatomy of HIS paper. (b) Contact angle performance on hydrophobic and hydrophilic regions. (c) Photographic image of the foldable HIS paper [52]. (**C**) Cotton-based innovative material for collecting sweat [59]. (**D**) Cloth-based chip and the extended WCECS wearable device [60]. (**E**) Janus fabric-based innovative material for collecting sweat [61]. (**F**) Janus silk for collecting sweat: (a) Schematic diagram of Janus silk on the skin surface. (b) The tests of transport on different fabrics [56].

Paper, despite its low cost and accessibility, may not provide the required level of mechanical stability and electrical conductivity for advanced wearables. To address the limitations, researchers developed some novel materials, which modified paper to meet the needs of sweat-collecting materials. For instance, Cheng et al. developed a paper-based microfluidic sweat sensor featuring a 3D origami structure (Figure 2A). To enhance performance, the researchers utilized wax dams to modify the hydrophilic properties of the thread-based channels. The sensor featured different detection strategies such as enzymatic reactions, pH indicators, complexes, and molecularly imprinted polymers (MIPs) on the surface of colorimetric and electrochemical electrodes. On-body experiments confirmed the reliability of the proposed sweat sensor and its potential for the non-invasive identification of various biomarkers found in sweat [55]. Moreover, in the work of Li et al., a highly integrated sensing paper (HIS paper) was constructed through a straightforward printing process. This HIS paper was fabricated by hydrophobic protecting wax, conducting electrodes, and MXene/methylene blue (Ti_3_C_2_T_x_/MB) active materials (Figure 2B). An advantageous feature of the HIS paper is the independent 3D positioning of the three-electrode configuration, which allows for easy enzyme decoration and fixation, as well as accessibility to electrolytes. Additionally, a dual-channel electrochemical sensor on the HIS paper was fabricated, capable of the simultaneous detection of glucose and lactate. The sensor exhibited sensitivities of 2.4 nA μM^−1^ and 0.49 μA mM^−1^, respectively. Overall, the HIS paper offers a miniaturized, low-cost, and flexible solution for various biochemical platforms, including wearable bioelectronics [52].

Further, cotton fabric, with its hypoallergenic properties and good moisture-wicking capabilities, can be a suitable choice for reusable wearable devices. For example, Criscuolo et al. utilized cotton fluidics sweat sampling to continuously deliver fresh sweat to the sensing area while simultaneously discarding previously tested samples (Figure 2C). The sensing system was multifunctional with the ability to collect sweat, transfer sweat, and detect various ions in sweat sample. One-step electrodeposited platinum nanostructures were the basic materials in the sensing system, which ensured reproducibility and biocompatibility. It successfully detected Li^+^ for Therapeutic Drug Monitoring (TDM) in psychiatric disorders, Pb^2+^ for monitoring heavy metal contamination, and K^+^ and Na^+^ for sports tracking. This biocompatible and efficient platform holds the potential for broader applications, allowing the measurement of numerous other biomarkers directly on the skin, which significantly advances the field of wearable device development [59].

As for fibers, especially those engineered to have specific properties, can be employed to create breathable textile structures, enhancing the comfort of wearable devices. In the study by Zheng et al., an innovative and flexible three-dimensional (3D) wearable electrochemical (EC) sensor for glucose analysis in sweat was presented, known as the wearable cloth-based EC sensor (WCECS) (Figure 2D). This WCECS features well-designed sweat collection channels and a cloth-based sweat transport channel. Notably, the capillary force drives the movement of sweat into the cloth-based chip, allowing for easy and cost-effective fabrication using screen-printing technology. The WCECS exhibited reliable performance in measuring glucose concentrations in sweat within the range of 0.05–1 mM, with a sensitivity of 105.93 μA mM^−1^ cm^−2^. This kind of sweat device has great benefits for wearable devices [60]. Based on the fibers and fabric materials, sweat collection devices have also been developed through material modification and structural design, such as the Janus structure. For instance, a novel all-fabric-based sweat-sensing platform that incorporates a redesigned directional sweat transport window was presented in the work of Peng et al. to enable the continuous transfer and monitoring of sweat. The directional sweat transport window is constructed by integrating hydrophobic and hydrophilic Janus wettability on both sides of a highly hydrophobic cotton fabric, which is then sandwiched between hydrophilic cotton layers (Figure 2E). This unique design allows sweat collected by the inner cotton layer from the area encompassing the window to be spontaneously and directionally transferred from the hydrophobic side to the hydrophilic side, even when the hydrophobic side faces downward, disregarding the influence of gravity. The outer cotton layer plays a dual role: drawing sweat away from the hydrophilic side of the directional sweat transport window through continuous evaporation and serving as a reservoir for sweat residue. Additionally, fiber-shaped sensing electrodes are affixed to the hydrophobic side of the directional sweat transport window. To demonstrate the feasibility, on-body and wireless monitoring of Na^+^, K^+^, NH_4_^+^, and pH levels was conducted in the sweat of an exercising volunteer. This directional sweat transport window approach presents a promising platform technology for various wearable applications [61]. Additionally, Zhang et al. presented a novel flexible and wearable sweat sensor made from fabric, capable of simultaneously detecting Na^+^, pH, and glucose. The sensor achieves high-efficiency collection of sweat through the use of a Janus fabric (Figure 2F). One side of the fabric is treated to be super-hydrophobic, while the other side is treated to be hydrophilic. This design enables the directional transport of sweat and the integration of multi-component detection functionalities. The resulting Janus fabric effectively transfers sweat from the skin side to the electrode, allowing for micro-volume collection as small as 0.2 μL. It also has good flexibility and comfortable wearability because of the soft fabric materials [56]. The Janus design principle is pivotal in materials science, frequently leveraged in the innovation of a broad spectrum of novel substances to enhance their functionality and practical outcomes.

**Figure 3:**
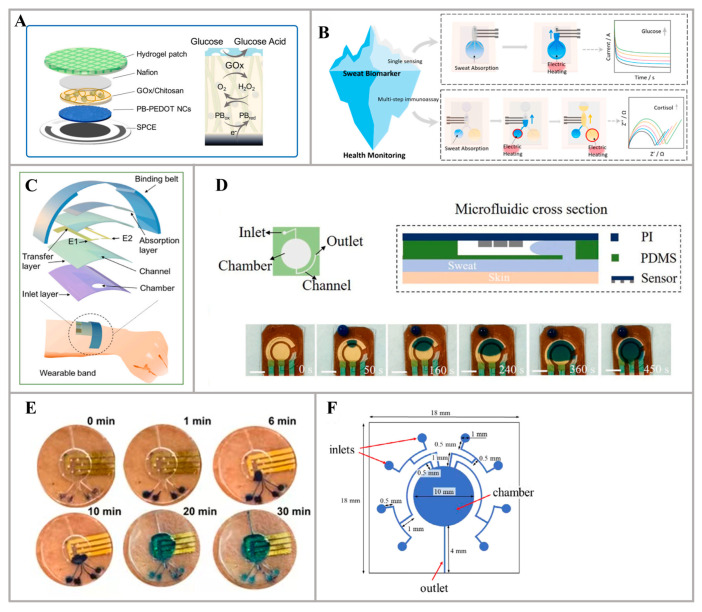
Structures of sweat collection devices of different materials: (**A**) Schematic of the multiple layers in the enzymatic electrode and the GOx working mechanism [62]. (**B**) Hydrogel-based innovative material for collecting sweat and biomarker detection [63]. (**C**) Explored view of the multilayer epidermal microfluidic device and its wearable platform [65]. (**D**) Schematics of the microfluidic chip and the tests of fluid flowing through the chamber [66]. (**E**) Photographs of sweat filling the microfluidic reservoir with time lapses [68]. (**F**) Structure design of the microfluidic system [69].

Different from the previous materials, hydrogels, with their high water content, can efficiently absorb sweat, making them suitable for wearables that measure hydration levels. However, they require specific storage conditions and are susceptible to mold growth, which can increase manufacturing and maintenance costs. For example, Lin et al. introduced a non-invasive sweat glucose sensor that utilizes hydrogel patches for the rapid sampling of natural perspiration, without the need for external activities to induce sweating. The sensor includes many layers, from the bottom to top, including Prussian blue and poly(3,4-ethylenedioxythiophene) nanocomposites (PB-PEDOT NC) (0.62 μm) as the mediator conducting layer, a glucose oxidase (GOx) immobilization layer, a Naifion protection layer, and a hydrogel sampling layer (Figure 3A). When attached to the biosensor, the GOx layer produces hydrogen peroxide (H_2_O_2_) in proportion to the glucose concentration in the patch, and the H_2_O_2_ is rapidly catalyzed and reduced through PB at a low overpotential. The optimized surface of the PB-PEDOT NC electrode significantly enhances non-invasive sweat glucose measurements, demonstrating excellent stability and electrocatalytic activity. Consequently, the GOx/chitosan/@PB-PEDOT NCs sensor exhibits a high-performance linear range of 6.25 μM to 0.8 mM and a sampling time of 15 min [62]. Additionally, in the work by Chen et al., a wearable sweat bioanalysis platform using a hydrogel-based system was created. The platform utilizes thermoresponsive hydrogels capable of absorbing natural sweat and releasing it through electric heating. The core component of the hydrogel system is a thermoresponsive poly(n-isopropyl acrylamide) (pNIPAM) polymer, which exhibits a lower critical solution temperature (LCST). When the temperature is below the LCST (42 °C), the hydrogel gradually absorbs sweat that is secreted from the skin. Once the temperature rises above the LCST, the hydrogel undergoes a phase transition, enabling the release of the collected sweat or preloaded reagents into a microfluidic channel for bioanalysis (Figure 3B). By employing this sweat-collecting device, a glucose sensor successfully realized real-time monitoring [63].

Microfluidic chips, offering high precision and tailoring possibilities, are best suited for laboratory settings where precise measurements are essential, although they are typically more expensive and complex to fabricate. For instance, a wearable microfluidic-based electrochemical sensor was proposed in the study by Xu et al. to enable the accurate and sensitive detection of uric acid (UA) in human sweat. The sensor comprises two layers, with the bottom layer being a microfluidic device fabricated using PDMS and the top layer consisting of a CPE (carbon-paste electrode) modified with a PEDOT:PSS hydrogel. The fabrication process yields a flexible sensor with exceptional sensitivity of 0.875 µA µM^−1^ cm^−2^ and a low detection limit of 1.2 μM (S/N = 3). Furthermore, the linear range of the sensor spans from 2.0 to 250 µmol L^−1^. The obtained results demonstrate good agreement with those obtained through an enzyme-linked immunosorbent assay (ELISA). Consequently, it is noteworthy to highlight the potential suitability of the PEDOT:PSS hydrogel-modified electrochemical sensor for monitoring UA concentrations in human biofluids [64]. Additionally, Wang et al. introduced a novel wearable sweat sensor platform that enables the simultaneous and calibration-free measurement of sweat rate and total electrolyte concentration. The platform utilizes a fluidic-controlled microfluidics design by PDMS, which facilitates the detection of sweat in a dropwise manner while mitigating the issues related to the dilution and mixing of old and new sweat samples (Figure 3C). Besides that, this design aligns with the decoupling principle by fixing one of the variables (concentration or volume) to create a univariate analysis system. The proposed microfluidic-based impedimetric sweat sensor device incorporates a short vertical channel (approximately 0.5 mm), a pair of embedded conductance electrodes, and an absorption layer, thus deviating from conventional approaches. Notably, the sensor demonstrates a detection range of approximately 0.5–20 μL min^−1^ cm^−2^ for sweat rate and 1–200 mM for total electrolyte concentration, which effectively covers the typical range of exercise-induced sweat [65]. In addition, Xiao et al. developed a non-invasive wearable electrochemical sensor based on uircase@MAF-7 that enables the accurate and sensitive detection of uric acid (UA) levels in sweat. This sensor combines a flexible microfluidic chip and a wireless electronic readout device (Figure 3D). The flexible microfluidic chip, fabricated using PDMS, facilitates the convenient and efficient collection of sweat samples. By encapsulating uricase, the MAF-7 material ensures the protection of enzyme activity. Consequently, the uricase@MAF-7-based electrochemical sensor achieves the highly sensitive detection of UA within a concentration range of 2 μM to 70 μM, with an impressive detection limit as low as 0.34 μM [66]. Furthermore, an innovative and cost-effective wearable patch utilizing microfluidic technology was successfully developed in the work of Nah et al. to non-invasively measure cortisol biomarkers in sweat. The patch design involved the utilization of PDMS as the substrate and microchannel fabrication material, while the electrodes were intricately crafted using laser-burned graphene (LBG). Besides that, the electrical properties were enhanced by incorporating Ti_3_C_2_T_x_ MXene. A remarkable achievement was observed when the cortisol antibody was meticulously combined with Ti_3_C_2_T_x_ MXene, resulting in a cortisol sensor that exhibited exceptional performance. The Ti_3_C_2_T_x_ MXene/LBG @ cortisol antibody sensor displayed an impressive linear range of 0.01–100 nM and a limit of detection as low as 1 pg/mL. These findings provide conclusive evidence that the developed strategy is highly suitable and conformable for rapid cortisol biomarker detection in point-of-care settings [67]. Moreover, in the study by Singh et al., a wearable, non-invasive biomolecular sensor was presented, enabling the monitoring of cortisol levels in sweat. The detection of cortisol is achieved through an electrochemical sensor that is specifically functionalized with a pseudoknot-assisted aptamer. This sensor is accompanied by a flexible microfluidic sweat-sampling system, ensuring efficient and rapid sweat collection while effectively segregating old and new sweat. Fabricated from PDMS, the skin-worn microfluidic sampler plays a vital role in providing accurate sweat analysis while maintaining a continuous monitoring capability for temporal changes (Figure 3E). Moreover, this aptamer is designed to be regenerable, facilitating continuous monitoring. To further enhance sensitivity and minimize background noise, an engineered pseudoknot is incorporated into the aptamer, effectively constraining it to operate in only two states. This novel approach significantly boosts the sensor’s overall sensitivity, enabling the precise detection of cortisol in artificial sweat solutions across a wide range, from 1 pM to 1 μM. Remarkably, the sensor exhibits an exceptional limit of detection (LOD) of 0.2 pM and demonstrates good linearity (R^2^ = 0.98). Additionally, the sensor demonstrates both high specificity and reproducibility, affirming its robust performance [68]. Additionally, Shitanda et al. successfully created a sensing system, incorporating an integrated microfluidic device made of PDMS that enables continuous monitoring of lactate levels in sweat (Figure 3F). The system encompasses a screen-printed electrode based on a graft-polymerized MgO-templated carbon (GMgOC) material, which was modified with LO_x_. The working electrode material of the screen-printed sensor was grafted polymerized MgO-templated carbon, while the substrate used was PI. The sensor exhibits outstanding performance with a linear range of 0 to 10 mM and a sensitivity of 36.2 μA/cm^2^/mM. The pliable nature of PDMS serves to prevent any bending of the sensor chip when the sensing system is affixed to mildly curved body regions, including the upper torso (front or back) or upper limbs (arms or legs) [69].

**Figure 4:**
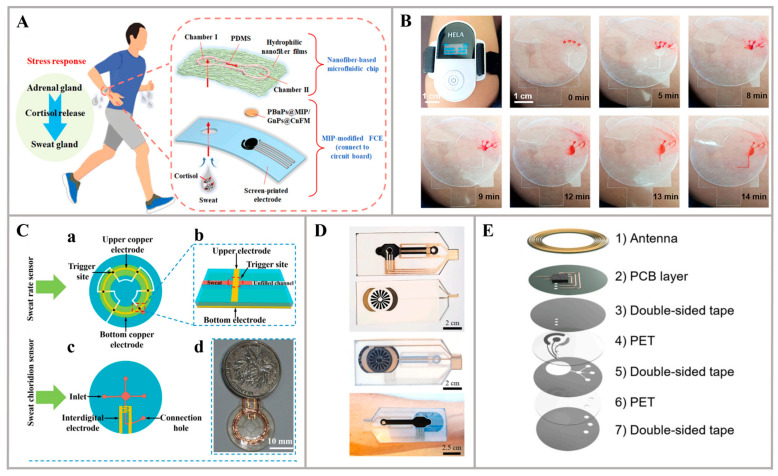
Structures of microfluidic devices: (**A**) Schematic representation of the layered sensor with a nanofiber-based microfluidic chip [70]. (**B**) Photographs of sweat flowing through the microchannel [53]. (**C**) Illustration of the PET-based microfluidic devices: (a) Top view of sweat rate sensor. (b) Sweat can wet two electrodes simultaneously (c) Top view of sweat chloride ion sensor. (d) Comparison of the size between sweat sensor and a 1-yuan coin [71]. (**D**) Schematics of the assembled wearable device [51]. (**E**) Explored view of the proposed glucose tag [72].

In addition to making microfluidic channels using PDMS, the scientists also used other materials to make microfluidic devices. For instance, in the work of Mei et al., a wearable electrochemical sensor was developed, using a microfluidic technique involving nanofibers to collect sweat and a molecularly imprinted polymer (MIP) for in situ and real-time sweat analysis. The sensor, with polyethylene terephthalate (PET) as the substrate, comprises two layers, a lower layer consisting of an MIP-modified electrode for sensing, and an upper layer involving a nanofiber-based microfluidic layer responsible for spontaneous sweat pumping. By electro-polymerization, the reported sensor with flexible polyethylene terephthalate (PET) as a substrate was composed of a PBnPs@MIP/GnPs@CnFM-modified FCE for electrochemical sensing and a nanofiber-based microfluidic chip for sweat extraction (Figure 4A). Consequently, the PBnPs@MIP/GnPs@CnFM/FCE sensor demonstrated a wide detection range of 1 nM to 1 μM, exhibiting excellent selectivity and stability when detecting cortisol as the model analyte. This integration of a molecularly imprinted electrochemical sensor with a nanofiber-based microfluidic chip represents a significant advancement in achieving the in situ and real-time monitoring of sweat cortisol [70]. Furthermore, Sun et al. developed a microfluidic patch using the “cut-and-paste (CAP)” method, employing a polydimethylsiloxane (PDMS) and polyethylene terephthalate (PET) film for patterning and transfer (Figure 4B). These newly proposed wearable sweat patches enable the real-time monitoring of glucose and lactate levels in sweat. The design, resembling a bear, consists of four polymer-based layers that can be automatically carved within 2 min using a desktop cutting machine. The sensing electrode of the patch is constructed with gold nanoparticles (AuNPs) and respective enzymes. A calculated concentration ratio of over 512.37 between blood glucose and iontophoresis-induced sweat glucose was observed, with the latter showing a delay of over 70 min after the peak concentration of the former. The fabricated sensors were employed to analyze and compare glucose and lactate levels in both exercise- and iontophoresis-induced sweat. These findings highlight the potential of the CAP method in developing wearable microfluidic platforms with low cost, versatility, and mechanical flexibility. This advancement paves the way for the development of wearable sweat microfluidic devices and their applications in individualized health monitoring [53]. In addition, a cost-effective and easily manufacturable microfluidic patch was presented in the study by Wei et al., capable of accurately measuring sweat rate and sweat chloride concentration in real time. The microchannel, with a width of 500 µm, was fabricated using laser cutting techniques. Subsequently, the microchannel layer was transfer-printed from a silicone board to a PET film (Figure 4C). The successful transfer printing of the electrode or microchannel layer was attributed to the strong adhesion between the PET film and the transferred layers. The microfluidic device was designed and manufactured using laser cutting technology and transfer printing technology, making the entire process simple and efficient. The stability and reliable sensing properties of this microfluidic patch on skin contact give it the potential for personalized medical applications [71]. Moreover, a wearable and fully printed microfluidic nanosensor in the work of Yang et al. was demonstrated, capable of measuring sweat rate, conductivity of sweat, and copper levels in sweat. The wearable system incorporates a PET inkjet-printed microfluidic component, enabling active sampling through reverse iontophoresis and the measurement of sweat volume/rate. To facilitate the reuse and continuous monitoring concept, the microchannel is emptied through sweat absorption by a sponge placed in contact with the air at the device outlet, causing the sweat in the sponge to evaporate (Figure 4D). The copper detection system exhibits a limit of detection of 396 ppb, a linear range extending up to 2500 ppb, and a sensitivity of 2.3 nA/ppb. With its strong interference resilience and extended shelf life, this system shows great potential for practical applications and continuous monitoring [51]. Additionally, in the work by Mirzajani et al., a microfluidic channel was fabricated, utilizing PET and double-sided tape to develop a channel with the thickness of 170 μm for sweat sampling (Figure 4E). The researchers developed an ultra-compact and wireless tag for battery-free sweat glucose monitoring, measuring only 1.2 cm^2^ in footprint and weighing 0.13 g. By employing NFC-based wireless power transmission and a compact antenna, the device operated without the need for batteries. And the working electrode consisted of a glucose oxidase/chitosan/AuNPs@Prussian blue/carbon electrode. As a result, the proposed sensor exhibited exceptional operating characteristics, including a limit of detection (LOD), limit of quantification (LOQ), and sensitivity of 24 μM, 74 μM, and 1.27 μA cm^−2^ mM^−1^, respectively. Through in vitro and in vivo experiments, the device successfully demonstrated its ability to attach to the body, collect sweat, and measure glucose, generating satisfactory results [72].

In summary, the impact of different materials on flexible and wearable devices is multifaceted, such as budget, robustness, skin-friendliness, and the desired end-use of the sweat sample. Therefore, the selection of materials should be made considering the specific requirements and constraints of each application.

### 2.2. Innovative Materials for Sweat Detection

In the burgeoning era of wearable technology, the substrates for electrochemical sensors are diversifying, with particular emphasis placed on those with enhanced flexibility [57,73,74].

**Figure 5:**
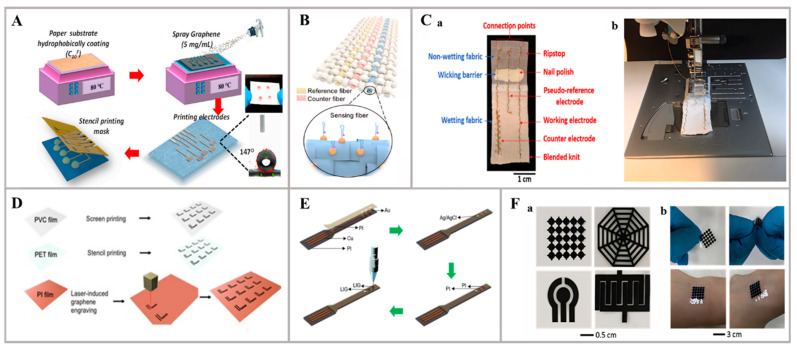
Innovative materials of sensing substrates: (**A**) The fabrication of ion-selective electrodes on a paper-based sensing substrate [75]. (**B**) The development of a functional wearable electrochemical cytokine sensor using a fabric-based sensing substrate [76]. (**C**) Figures showing the stitched sensing devices based on a fabric sensing substrate: (a) a single completed device; (b) the way the devices are stitched using a sewing machine [77]. (**D**) Fabrication steps employing PVC/PET/PI film as the sensing substrate [78]. (**E**) Illustration of screen printing and laser-induced graphene composite electrode [79]. (**F**) Sensing substrate of porous CNT-EVA film: (a) structures fabricated from free-standing CNT-EVA films by a laser-cutter; (b) photos showing the flexibility and adhesion of as-prepared structures for wearable bioelectronics [34].

Paper and textile substrates stand out for their distinctive attributes, which are notably lightweight and affordable, facilitating scalable manufacturing processes. Moreover, their breathability and moisture-wicking qualities promote skin health by allowing for natural perspiration, thus minimizing irritation. Because of the above merits, researchers have modified these materials to function as the substrate for detection electrodes. For instance, An et al. devised a novel paper-based, flexible, all-solid-state, multichannel ion-selective electrode (ISE) for the real-time analysis of ions in human sweat. This integrated solid ISE consists of four channels capable of simultaneously detecting K^+^, Na^+^, Cl^−^, and pH with remarkable precision, dependable potential stability, and low detection limits. To further enhance the sensor’s potential stability, a high-quality graphene suspension was utilized as an ion-to-electron transducer. Additionally, by modifying the super-hydrophobic paper matrix with a fluorinated alkyl silane, the water-layer effect was minimized, thereby improving the overall sensor performance (Figure 5A). This research highlights the promise of modified paper-based substrates for wearable electrochemical sensors, owing to their exceptional flexibility and conductivity [75]. In addition, in the work of Chu et al., a novel fabric wearable system was proposed for the sweat detection of low-concentration IL-6, a typical cytokine. This system integrates a newly developed sensing fiber network with commercially available textile materials, resulting in a flexible and wearable electrochemical fabric. The fabric incorporates a carbon nanotube/graphene (CNT/G) composite fiber functionalized with an IL-6 aptamer; thus, it offers excellent flexibility, anti-fatigue properties, and breathability, which has not been reported previously (Figure 5B). Moreover, the integrated fabric demonstrates the capability to detect IL-6 in the range of 1 pg mL^−1^ to 100 ng mL^−1^, with an impressive limit of detection (LOD) of 280 fg mL^−1^, significantly lower than that reported for exocrine biofluids. Overall, this aptamer-based fabric wearable system, by combining a new sensing fiber network with commercially available textile materials, holds great potential in various fields such as sports medicine, military applications, and healthcare [76]. Furthermore, an inexpensive (0.22 USD/sensor) and disposable wearable biosensing platform was introduced in the study by Piper et al., integrating conductive gold-coated threads into fabrics using a stitching technique (Figure 5C). This platform is equipped with thiolate self-assembled monolayers capable of detecting a wide range of biomarkers. The all-textile sensing platform incorporates Au plasma-coated multifilament threads that are stitched into Ripstop fabric and an elastic blended knit, allowing for a stretchability of up to 150% without compromising the observed electrochemical response. Moreover, the devices can detect glucose in human sweat effectively and continuously within the clinically relevant range of 0.1–0.6 mM. Notably, the sensors exhibit a sensitivity of 126 ± 14 nA/mM of glucose and a limit of detection of 301 ± 2 nM [77].

Polymers are also commonly used as substrates because of their robust mechanical integrity, resistance to chemicals, and electrical insulating properties. Commercial screen-printed electrodes (SPEs) often employ polymers like PET, PI, or PVC, thanks to their transportability, economic efficiency, and capacity for large-scale fabrication. For example, Teekayupak et al. developed a sensor array capable of simultaneously detecting K^+^, Na^+^, and Ca^2+^ ions in sweat. The fabrication of ion-selective electrodes (ISEs) was achieved through large-scale manufacturing techniques. To achieve mass manufacturing, the authors utilized polyvinyl chloride (PVC), polyethylene terephthalate (PET), and polyimide (PI) substrates for ISE fabrication using stencil printing, screen printing, and laser engraving methods, respectively (Figure 5D). To optimize the sensitivities of the electrodes, the surfaces were modified with various carbon nanomaterials, such as multi-walled carbon nanotubes (MWCNTs), graphene (GR), carbon black (CB), and mixed suspensions of these materials, forming an intermediate layer. To streamline the fabrication process, an automated 3D-printed robot was employed for the drop-casting procedure, eliminating the need for manual steps. The sensor array was further optimized, resulting in detection limits of 10^−5^ M, 10^−5^ M, and 10^−4^ M for K^+^, Na^+^, and Ca^2+^ ions, respectively. This developed sensing platform provides a low-cost solution for electrolyte detection, suited for point-of-care applications [78]. Moreover, in the work by Chen et al., a wearable sweat sensor was introduced, capable of simultaneously and separately detecting UA, AA, and Tyr. This innovative sensor combines the benefits of screen-printed electrodes (SPEs) and laser-induced graphene (LIG) electrodes (Figure 5E). The supporting substrate of the SPEs is made of polyimide (PI) due to its exceptional mechanical properties, while the conductive path is created using Cu to address the inherent brittleness of carbon materials. The integration of LIG, which offers a large specific surface area and excellent electron transfer properties, enhances the sensor’s electrochemical activity and sensitivity. Additionally, by using SPEs with excellent mechanical properties, the sensor achieves high flexibility and remarkable structural stability, which maintains an 88.1% UA detection performance after 20,000 bends [79]. In addition to the common commercial polymer substrates, there are also synthetic new copolymer substrates. A mediator-free wearable electrochemical biosensor was fabricated in the study by Xia et al. for real-time sweat glucose monitoring by constructing a flexible and hierarchically structured meso/macro-porous film composed of carbon nanotubes (CNTs) and ethylene–vinyl acetate copolymer (EVA). The functionalized CNT-EVA film allows for direct electron transfer-based bioelectrocatalysis, eliminating the need for any mediators, in the glucose oxidase (GOx)–horseradish peroxidase (HRP) bienzyme system for glucose monitoring (Figure 5F). The biosensor exhibits an exceptional sensitivity of 270 ± 10 μA mM^−1^ cm^−2^ and a limit of detection (LOD) of 3 μM. Additionally, the high electrical conductivity and skin conformability of the flexible CNT-EVA film enable its application in monitoring surface electromyography (sEMG) with high signal-to-background sensitivity. This film holds great promise for the development of more efficient, accurate, and cost-effective biosensors not only for glucose monitoring but also for other biochemical molecules [34].

**Figure 6:**
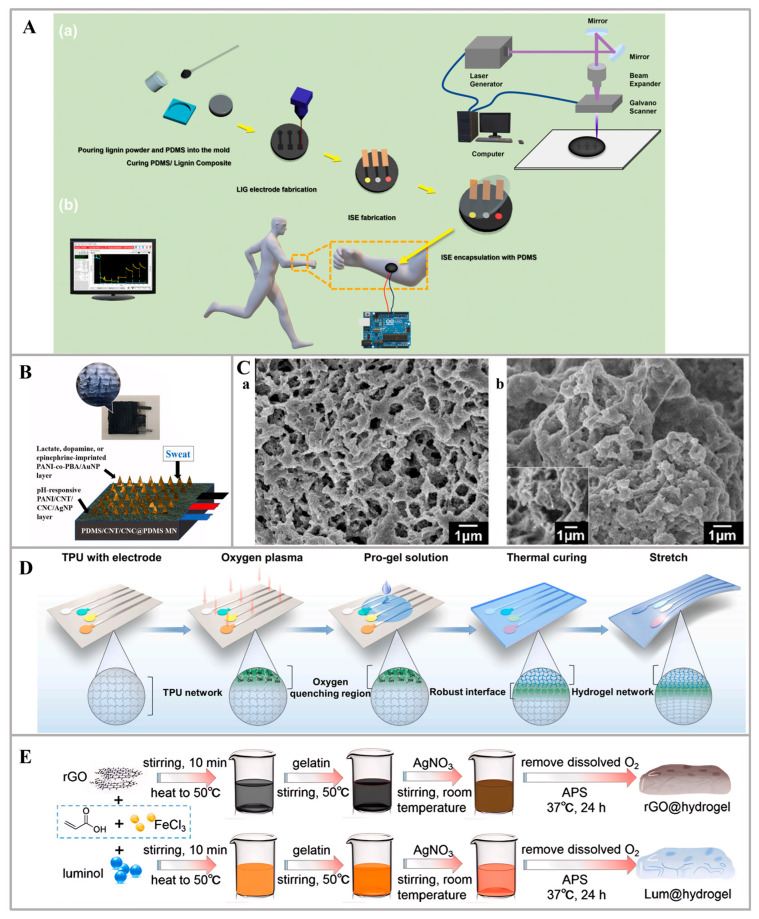
Sensing substrate of PDMS and hydrogel: (**A**) Schematic image of the fabrication process of the LIG-based sensing platform: (a) The fabrication process of the LIG-based sensing platform. (b) The sensing platform attached to the wrist and its sensing results [80]. (**B**) The fabrication of wearable electrochemical sensor patches on a polydimethylsiloxane (PDMS)-based sensing substrate [81]. (**C**) Characterization of the hydrogel sensing substrate: (a) SEM micrograph of the TA-Ag-PANI hydrogel and the TA-Ag-CNTPANI hydrogel; (b) inset: the section of the TA-AgCNT-PANI hydrogel [82]. (**D**) The fully integrated hydrogel elastomer-based sensing substrate [83]. (**E**) Detailed preparation procedures for hydrogels as a sensing substrate using a simple one-pot synthetic approach [35].

Additionally, innovative flexible materials to fabricate microfluidics, such as PDMS and hydrogels, are opening new frontiers in wearable sensor design. PDMS boasts superior elasticity and flexibility, accommodating significant deformation without compromising the sensor’s integrity. For instance, Lee Et al. proposed a sensor, fabricated by an elastomer substrate, which was made by mixing polydimethylsiloxane (PDMS) and lignin in specific weights. Laser-induced graphene (LIG) was formed on the PDMS/lignin composite substrates using Laser Direct Writing (LDW) to create the sensor (Figure 6A). After undergoing laser irradiation, the PDMS/lignin composite transformed into a porous structure, which is crucial for the sensor’s optimal functionality. To assess the performance of the sensor, sodium and potassium ion-selective electrodes (ISEs) were fabricated alongside an Ag/AgCl electrode on the PDMS/lignin composite. These electrodes were utilized to measure the potential difference at various concentrations of Na^+^ (10^−1^–10^−7^) and K^+^ ions (10^−1^–10^−8^). The results exhibited remarkable sensitivity values of 63.6 mV/dec (Na^+^, *n* = 6) and 59.2 mV/dec (K^+^, *n* = 7) for the fabricated sodium and potassium ion-selective electrodes, respectively. These sensitivity values closely adhere to Nernstian behavior, with correlation coefficients (R^2^) of approximately 0.99988 and 0.99855, respectively. Importantly, the sensor demonstrated cyclic stability, enduring 5000 cycles, and exhibited a Gauge Factor of approximately 20. The sensor also demonstrated promising potential of motion monitoring capabilities, enabling the detection and tracking of muscle contractions and pulse waves [80]. In addition to the application of the same material, PDMS, for microfluidics, there are some innovative structure designs, like layer-by-layer construction. In the study by Mugo et al., a novel wearable platform was presented. It consists of molecularly imprinted microneedles for the simultaneous electrochemical detection of pH, epinephrine, dopamine, and lactate biomarkers in human sweat. The sensors were constructed through layer-by-layer (LbL) assembly on a polydimethylsiloxane (PDMS) substrate that was coated with a conductive composite of PDMS, carbon nanotubes (CNTs), and cellulose nanocrystals (CNCs) (PDMS/CNT/CNC@PDMS). The pH sensor utilized a pH-responsive composite layer of polyaniline (PANI), CNTs, CNCs, and silver nanoparticles (AgNPs), while the epinephrine, dopamine, and lactate sensors incorporated an additional layer of PANI-co-3-aminophenylboronic acid (PBA) imprinted with epinephrine, dopamine, or lactate, respectively, along with gold nanoparticles (AuNPs), on top of the PANI/CNT/CNC/AgNP composite layer (Figure 6B). Incorporating conductive materials onto the PDMS substrate shows promising potential for enhancing sensor performance. Moreover, by modifying the template molecule during fabrication, the biomarker target on the PANI-co-PBA/AuNP sensing platform can be easily adjusted to meet various requirements [81]. Moreover, researchers have also advanced the flexible field by innovating conventional hydrogel systems, resulting in the development of wearable sensor substrates. A wearable sweat sensor was developed in the work by Xu et al., utilizing a multifunctional conductive hydrogel for the on-body detection of pH and tyrosine (Tyr). The hydrogel composite consists of PANI hydrogel decorated with tannic acid chelated-Ag nanoparticles (TA-Ag NPs) and carbon nanotubes (CNTs). The PANI hydrogel, with its large specific surface area, exhibits high catalytic activity for Tyr and pH detection (Figure 6C). The introduction of TA-Ag NPs ensures efficient antibacterial activity of the hydrogel, making it suitable for application on the skin surface. Additionally, the incorporation of CNTs enhances the hydrogel’s conductivity and improves its overall catalytic and mechanical properties. This electrochemical sensor, based on the TA-Ag-CNT-PANI composite hydrogel, holds significant potential for personalized healthcare, indicating a promising approach in the design of wearable sweat sensors [82].

Owing to the substantial water content, biocompatibility, and tunable mechanical characteristics, hydrogel materials are also widely applied in the wearable non-invasive electrochemical sweat sensors towards biomedical applications. A lot of efforts have been done to modify the hydrogel materials to further improve their performance. For instance, Wang et al. have successfully developed a skin-like hydrogel elastomer-based electrochemical device for the comfortable real-time detection of pH, Na^+^, and K^+^ in sweat by integrating hydrogels with a thin TPU film with conductive layers. By bridging the hydrogel and conductive material through a TPU film, the device maintains the mechanical softness of the hydrogel, the conductivity of the electrode material, and a Young’s modulus similar to that of the skin (Figure 6D). This unique combination ensures compatibility with the skin interface and provides a comfortable wearing experience. Remarkably, it maintains excellent electrochemical performance even when subjected to a 30% strain, eliminating the need for complex external structural designs. The simplicity of fabrication, along with the combined advantages of hydrogels and conductive materials, makes this device highly promising for real-time biofluid monitoring with comfort and mechanical flexibility [83]. Additionally, in the study by Hu et al., a versatile sensing platform was introduced, utilizing hydrogels modified with reduced graphene oxide (rGO) and luminol (Lum) to enable motion monitoring and sweat analysis (Figure 6E). This platform comprises a strain-sensing unit and a sweat detection unit, allowing for the simultaneous analysis of multiple physiological markers present in sweat. By leveraging the inherent flexibility, adhesiveness, and self-healing properties of hydrogels, in conjunction with the closed bipolar electrode (c-BPE) system, an economical, user-friendly, and biocompatible sensing platform has been established, bridging the gap between versatile system aspirations and the challenges of cumbersome design. The working electrode is composed of a luminol-functionalized hydrogel (Lum@hydrogel), which generates electrochemiluminescence (ECL) signals in response to analytes at the cathode. On-body experiments have demonstrated the platform’s rapid response to motion behaviors, as well as its exceptional performance in analyzing various physiological indicators (urea, lactic acid, and chloride ions) in sweat [35].

### 2.3. Innovative Materials for Sweat Sensor Self-Powering

In the realm of wearable electrochemical sweat sensors, self-powered operation is typically achieved through two distinct mechanisms. The first involves the conversion of mechanical energy into electrical energy, as exemplified by triboelectric nanogenerators (TENGs) and piezoelectric nanogenerators (PENGs). The second mechanism entails the transformation of chemical energy into electrical energy, which can be realized through redox reactions or biofuel cells [45,84,85]. The innovative materials for self-powering are various according to the different mechanisms.

**Figure 7:**
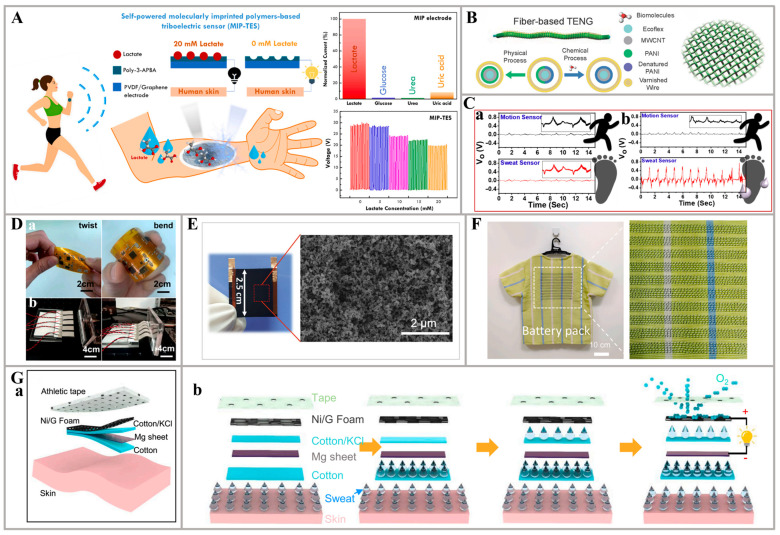
Innovative materials for self-powered sweat sensors: (**A**) Self-charging MIP-integrated triboelectric sensor for sweat lactate detection [36]. (**B**) Structure of fibrous TENG and its woven textile equivalent [86]. (**C**) Output voltage of the sweat sensors under different sweating conditions: (a) Without sweat condition. (b) With sweat condition [87]. (**D**) Depiction of PENG deformation test: (a) Picture of twist and bending. (b) Picture of bending test by a linear motor [88]. (**E**) The optical and SEM images of the self-powered biosensor [45]. (**F**) Photograph showing a T-shirt crafted from fabric activated by sweat-generated energy [91]. (**G**) Characteristics of the SABs: (a) schematic of the sweat-activated battery based on cotton; (b) working principle of the SAB through oxygen reduction processes [92].

Integrating self-powered devices with biosensing apparatus represents a principal design paradigm. The utilization of mechanical energy for electricity generation imposes stringent material requirements. For instance, triboelectric nanogenerators may suffer from material degradation due to repeated friction, affecting their lifespan. Conversely, piezoelectric nanogenerators necessitate the selection of sensitive materials with piezoelectric properties. These factors have prompted researchers to explore alternative approaches to power generation, focusing on chemically derived electricity. This method involves the generation of current through electron transfer, thereby facilitating the conversion of energy. For example, Kanokpaka et al. developed a self-powered triboelectric sensor utilizing molecularly imprinted polymers (MIPs) for non-invasive lactate detection in monitoring applications. The sensor incorporates a flexible electrode composed of free-standing PVDF/graphene, which is modified with poly (3-aminophenyl boronic acid) (3-APBA) to imprint lactate molecules, leading to changes in surface properties upon lactate adsorption (Figure 7A). By integrating the MIP lactate sensor with a triboelectric nanogenerator, the system efficiently combines the selective lactate detection capabilities with the mechanical energy harvested from contact and separation, ensuring an adequate power supply. The findings demonstrated that the higher TENG output signals of Voc and Isc from 20 to 30 V and 2.5 to 4.0 μA were investigated when decreasing lactate concentration from 20 to 0 mM. In the absence of lactate, all 10 LEDs illuminate, while only 2 LEDs light up when detecting a 20 mM lactate solution. This self-powered triboelectric lactate sensor effectively operates multiple LED lights without relying on an external energy source, highlighting its potential for practical implementation in wearable sensors for human skin [36]. Additionally, in the research by Zhao et al., a flexible fiber-based triboelectric nanogenerator (F-TENG) was fabricated, employing body motion to capture energy. This device is integrated with wearable biosensors for the real-time analysis of sweat. The F-TENG is constructed by layering multi-walled carbon nanotubes (MWCNTs) and polyaniline (PANI) onto an ultra-stretchable Ecoflex fiber and twining varnished wires (Figure 7B). It can convert mechanical energy from human motion into triboelectric sensory signals without requiring external power sources, thanks to the triboelectric effect. Moreover, the triboelectric output current of enzyme-modified F-TENGs is directly linked to the concentration of biomarkers, making it a reliable biosensor signal. The F-TENG can be seamlessly incorporated into clothing to create a self-powered closed-loop health-monitoring system [86]. Additionally, a textile-based wearable sweat sensor was presented in the work by Baro et al. on the principle of triboelectrification. By leveraging the interaction between hydrated salts found in human sweat and adsorbed molecules on the ZnO surface, the sensor enables the detection and measurement of sweat analytes. The authors grew ZnO on cotton fabric using a simple chemical technique and incorporated it into a wearable sweat sensor based on the STENG (Sweat Triboelectric Nanogenerator) concept. The active triboelectric layer of the STENG was constructed using optimized ZnO–Teflon (PTFE) nanorods on the cotton fabric. It was proposed that the attachment of hydrated Cl ions from saline water to physisorbed water molecules on ZnO enhances the n-type conductivity, thereby amplifying the triboelectric output (Figure 7C). The developed prototype demonstrated a sensitivity of approximately 0.02 V/µL and a detection limit of approximately 4.8 µL [87]. Furthermore, Li et al. reported a human-joint-enabled flexible and wearable self-sustainable sweat sensor patch (FWS^4^P) based on piezoelectric nanogenerators (PENGs). The sensor is equipped with a sweat-resistant self-sustainable energy supply and wireless communication interface. By utilizing a PENG integrated within the sensor, biomechanical energy from movable joints such as the finger, cubital fossa, and popliteal space is efficiently converted into electricity, enabling self-powering functionality (Figure 7D). The PENG is constructed using a layer-by-layer structure consisting of PET, Ag, and PVDF as the supporting substrate, electrode layer, and piezoelectric layer, respectively. Furthermore, the sensor is capable of detecting physiological parameters present in sweat, such as Na^+^, K^+^, and pH. These parameters are wirelessly transmitted to a user interface via Bluetooth communication. This groundbreaking system exemplifies the potential of wearable electronics driven by human joints, showcasing an effective self-sustainable energy supply and multiplexed physiological detection [88].

In addition to the self-powered technology that converts mechanical energy into electrical energy as mentioned earlier, researchers are also actively developing self-powered devices that can transform environmental thermal energy into electricity. For instance, Guan et al. presented a new self-powered wearable sweat lactate analyzer for collecting sports data. This device utilizes the sweat-evaporation-biosensing coupling effect, which includes a sweat evaporation biosensor, a sweat-collecting shell, a capacitor for storing power, a wireless transmitter for transmitting lactate analysis data, two cotton pads, and a Poly-dimethylsiloxane (PDMS) substrate for sweat flow. The analyzer’s self-powered biosensor is made from a porous carbon film that has been modified with lactate oxidase (Figure 7E). The hydrophilic carbon absorbs sweat and converts environmental thermal energy into electricity through sweat evaporation, and the generated voltage not only powers the device but also provides biosensing information. This research has the potential to inspire a new research direction in developing self-powered wearable exercise-analyzing systems, as well as contribute to the advancement of sports big data [45].

Biofuel cells constitute a class of devices that harness microbes or other biocatalysts to oxidize fuels into electrons and ions, which are subsequently passed through an electrolyte to drive an external circuit to generate an electric current. For example, Veenuttranon et al. reported a self-powered biosensor, driven by glucose, that relies on a single enzyme and utilizes screen-printable nanocomposite inks for the bioanode and biocathode. The anode ink is enhanced using naphthoquinone and multi-walled carbon nanotubes (MWCNTs), while the cathode ink is modified with a Prussian blue/MWCNT hybrid before being immobilized with glucose oxidase. The flexible bioanode and biocathode enzymatically consume glucose, resulting in an open-circuit voltage of 0.45 V and a maximum power density of 266 μW cm^−2^. This biosensor, when coupled with a wireless portable system, can effectively convert chemical energy into electric energy and detect glucose levels in simulated sweat samples. It demonstrates the capability to detect glucose concentrations up to 10 mM [48]. Furthermore, in the study by Sun et al., a novel flexible and wearable biofuel cell (BFC) was fabricated that harnessed the bioenergy from human sweat in real time. This groundbreaking device represents the first example of a flexible and wearable epidermal microfluidic BFC capable of simultaneously sampling sweat and generating bioelectricity on the skin of individuals who consume alcohol. The system consists of a microfluidic module that interfaces with the skin to facilitate the on-body transport, sampling, storage, and excretion of fresh sweat, as well as a BFC module that utilizes a flexible ethanol/oxygen BFC to generate non-invasive and real-time bioenergy. The utilization of high-flexibility and mass-productive substrates, such as PI and PET, in the microfluidic and BFC modules enables the epidermal ethanol BFC to be comfortably worn on different skin regions, ensuring effective bioenergy generation. The bioanodic catalyst, alcohol oxidase (AOx), facilitates the oxidation of ethanol biofuel in the presence of oxygen as the natural mediator, whereas the biocathodic catalyst, bilirubin oxidase (BOx), directly reduces oxygen. As sweat enters the system, the on-body epidermal ethanol BFC utilizes sweat alcohol and dissolved oxygen to generate electrical bioenergy in real time [89]. Moreover, an innovative system called a wearable sweat-based energy generator (SEG) was proposed in the study by Chen et al., which aimed to power a soft artificial muscle and establish a self-powered conjunct system. The SEG features a complementary radial-arrayed sector electrode structure, enabling it to simultaneously generate electricity from perspiration while also serving as an energy source for artificial muscles. The SEG consists of copper foil (cathodes) coated with single-walled carbon nanotubes (SWCNTs) and zinc foil electrodes (anodes). When moistened by sweat, which acts as an electrolyte, the SEG generates electric power by allowing ion flow between the anode and cathode. In this system, the copper foil coated with SWCNTs functions as the cathode, facilitating the reduction reaction (O_2_ + 2H_2_O + 4e^−^ → 4OH^−^), while the zinc foil acts as the anode, supporting the oxidation reaction (Zn − 2e^−^ → Zn^2+^). These spontaneous oxygen reduction reactions between the reaction electrodes and sweat drive the flow of electrons in the external circuit. The generator utilizes the redox reaction in sweat to generate electricity, achieving a maximum power density of 18.3 μW cm^−2^ at a resistance of 0.3 kΩ with a small amount of sweat (0.2 mL). Additionally, the sweat generator is integrated with an EMG sensor for detecting surface electromyography signals [90]. In addition to planar self-powered devices, researchers have also developed linear self-powered apparatuses. A sweat-activated yarn battery (SAYB) with a core–sheath structure was introduced in the research by Ju et al. In this design, the core consists of a Zn wire, the sheath is made of cotton yarn, and the outermost layer is composed of carbon yarn, serving as the anode, sweat-wicking separator, and cathode, respectively (Figure 7F). The carbon yarn can act as a metal-free electrocatalyst for oxygen reduction within the battery. During perspiration, the cotton sheath absorbs sweat, triggering oxidation (Zn – 2e^−^ > Zn^2+^) and reduction (O_2_ + 2 H_2_O + 4e^−^ > 4OH^−^) reactions at the anode and cathode. The thin and hydrophilic cotton sheath enables the rapid activation of the SAYB, requiring only 1 μL of 100 mM NaCl solution within 3 s for activation. This is possible due to the shortened distance and accelerated liquid penetration between the electrodes. The SAYB exhibits a high power density of 1.72 mW cm^−2^ and an energy capacity of 15.3 mAh, surpassing those previously reported for cotton-yarn-based sweat-activated batteries. Moreover, the authors successfully produced a large-scale SAYB measuring 60 m in length, which was utilized to create a sweat-activated energy fabric (5 m long and 0.5 m wide) for self-powered body motion sensing [91]. Additionally, Liu et al. reported a thin, flexible bandage that functions as a sweat-activated battery (SAB), delivering high energy capacity and power density. Comprising four layers, the topmost layer is a graphene-coated nickel (Ni/G) foam, followed by a cotton/KCl layer, a Mg sheet, and a bottom cotton layer that collects sweat (Figure 7G). The battery operates on a redox mechanism, utilizing sweat as the electrolyte. Sweat absorbed by the cotton layer dissolves the connecting chemical, KCl, activating the battery by linking the anode and cathode. The anode, Mg, is oxidized to Mg^2+^, producing electrons. O_2_ passes through the Ni/G foam cathode and reacts with H_2_O to reduce to OH^−^, simultaneously gaining double the amount of electrons. When connected in series, four SAB cells provide high power density to wirelessly power a skin-integrated electronic system with state-of-the-art wearable sensors for over an hour [92].

### 2.4. Others

Moreover, additional categories such as conductive ink and linear sensors are being utilized to form innovative materials that enhance the performance of sweat sensors. A surface strain-redistributed elastic fiber (SSRE-fiber) was fabricated by Wang et al. for monitoring sweat sodium levels. The sensor demonstrated exceptional stretchability and sensing stability. Its design incorporated a unique unilateral bead structure within the SSRE-fiber, resulting in significant changes to the surface strain distribution during deformation. The active sensing materials, strategically fabricated on the unilateral bead region with a high Young’s modulus, maintained their integrity even when stretched within the range of 0–200%. This pioneering SSRE-fiber platform paves the way for the design of highly stretchable and stable electrochemical sensors [93]. Additionally, another fiber-shaped organic electrochemical transistor (FOECT)-based sensor was reported in the study by Qing et al. This sensor exhibits exceptional transconductance (g_m_) and ultrasensitivity, enabling the detection of multiple biomarkers, including dopamine, lactate, and glucose. To achieve this, the FOECT was created by simultaneously synthesizing highly ordered polypyrrole (PPy) nanowires and porous PPy microflowers on a reduced graphene oxide (rGO)/cotton@polyethylene terephthalate (CPET) fiber. This innovative approach resolves the trade-off between achieving high charge carrier mobility and large volumetric capacitance for substantial g_m_. The resulting woven FOECT system can effectively monitor dopamine, lactate, and glucose with low detection limits (1 nM), high sensitivity (46.8 ± 3.3 mV/dec for dopamine, 85.3 ± 6.1 mV/dec for lactate, and 235.5 ± 20.8 mV/dec for glucose), good selectivity, and consistent results in artificial sweat. This research presents a practical strategy for the large-scale and stable fabrication of FOECTs, thereby driving advances in personalized healthcare sweat sensors [94]. Textile organic electrochemical transistors (txOECTs) were introduced in the study by Copped et al. They have been functionalized with ion-selective membranes. This advancement serves to enhance selectivity and offer non-invasive and wearable solutions for monitoring crucial physiological parameters in biofluids of clinical significance, such as sweat. By utilizing txOECTs, the process of acquiring samples is simplified, and the selective detection of specific ions, such as sodium, potassium, and calcium, in sweat is achieved. The sensor successfully demonstrates the selective response of the textile biosensors to sodium, potassium, and calcium ions, proving their ability to discriminate among the cations over the 10^−5^–1 M concentration range, a concentration range found in sweat. Importantly, the integration of txOECTs into the sensor design opens up possibilities for accessing physiological data that were previously inaccessible, thereby enhancing the monitoring of patients’ overall health [95]. A self-healing conductive ink was reported in the work by Son et al. It was developed by incorporating graphene (Gr) into a self-healing polymer called poly(1,4-cyclohexanedimethanol succinate-co-citrate) (PCSC) using a fluid dynamics process. The resulting PCSC/Gr (P-Gr) conductor demonstrated a high electrical conductivity of 1243 S m^−1^ and could withstand a tensile strain of 213%. The ink was easily screen-printed onto a flexible textile substrate to create a serpentine-structured electrode, which was successfully used to produce a wearable sensor for the electrochemical detection of Na^+^ in human sweat. Remarkably, the sensor’s sensitivity was shown to be restored during cut–heal cycling. This work opens up possibilities for the commercialization of devices that require both mechanical resilience and stable electrical performance, such as e-skin, sweat sensors, soft robotics, and biofuel cells [96].

## 3. Conclusions and Perspective

This article provides an extensive overview of cutting-edge research on innovative materials and their deployment in three key areas: sweat collection, detection, and self-powering. Notably, conductive materials (like graphene and nano metal composites) are popular innovative materials utilized for electrodes and powering, while flexible and thin materials (such as fabric, hydrogel, and PDMS) are applied for substrates and sweat collection as shown in Table 1. The sensors are always in the sensing layer and sandwiched by two layers of the substrate which isolate the sensor and circuit from human skin to avoid harm. Additionally, these substrates can also improve the deformability and stretchability of wearable sensors. A self-powered sweat sensor typically integrates its energy-harvesting component with the sensing element to create a standalone device capable of both generating its own power and continuously monitoring physiological parameters in real time, which are compared in Table 2. This integration allows for stable operation without the need for external power sources, making it highly suitable for long-term health-monitoring scenarios, especially during physical activities or sports events where continuous tracking is essential. In some works, there are also insulating layers between the sensing layer and substrates to support and insulate the sensing layer and active catalyst layer to improve the sensing performance of the sensing layer. 

The development of innovative material-based wearable non-invasive electrochemical sweat sensors represents a significant advancement in the field of biomedical applications. These sensors have the potential to revolutionize healthcare by providing real-time, accurate, and continuous monitoring of important biochemical parameters in sweat. The innovative materials used in sweat collection, sweat detection, and self-powering play a crucial role in the success of these sensors, ensuring comfort, accuracy, and sustainability. In the future, as the technology continues to evolve, it is exciting to imagine the potential applications and impact of these innovative material-based wearable electrochemical sweat sensors in the field of healthcare. Nonetheless, there is considerable scope for the advancement of wearable sweat-sensing technology if the field is to progress and facilitate tailored, smart medical treatments:

- Fabricating materials to ensure comfort, power efficiency, and sensitivity. Comfort and user experience are critical for long-term wearability, necessitating skin-friendly, non-irritating materials that do not disrupt daily activities. Additionally, power efficiency is equally important, with self-powered technologies requiring high efficiency and stable performance to sustain long-lasting operation without frequent charging or battery replacement. Moreover, sweat sensors with high sensitivity and specificity are essential, and capable of detecting a broad range of analytes in sweat. This requires the optimization of materials and detection mechanisms to enable the accurate differentiation and measurement of various biomarkers.
- Sensor performance in stability, durability, miniaturization, and multiplexed sensing systems. Wearable sensors, particularly those utilized in the biomedical field, are subject to stringent demands, primarily regarding their stability and durability. These devices must endure continuous operation without compromising their functionality due to environmental stressors such as varying temperatures, high humidity, and direct sunlight exposure. Furthermore, the challenge of miniaturization without sacrificing functionality is an area of active research. The ability to make sensors smaller while retaining their accuracy and reliability, coupled with their integration into a network of other wearable technology, is key to the future of wearable health monitoring. While miniaturizing, aiming to maximize its functionalities is the objective as well, which can detect different physiological indicators through a single sensor device.
- Personal sensing devices: customization, privacy, and validation. Customization and personalization are paramount due to the unique physiological responses that vary from person to person. Personal sensing devices must therefore be capable of accommodating personalized settings and algorithms to ensure that the information provided to the user is accurate and relevant. Additionally, data privacy and security are of utmost importance considering the sensitive nature of health data collected by these devices. Stringent encryption and robust data protection mechanisms are necessary to prevent unauthorized access and potential misuse of the information. Furthermore, it is essential to establish the accuracy and reliability of personal sensing devices in clinical settings to gain the trust of healthcare providers and enable widespread adoption in medical practice. This validation process involves rigorous testing and verification to ensure that the devices provide trustworthy results.

The aforementioned challenges and the corresponding potential solutions offer substantial opportunities for the ongoing advancement of wearable sweat sensors. Overcoming these hurdles requires a multidisciplinary effort involving material scientists, clinical researchers, and engineers. An ideal wearable sweat-sensing system should be highly compatible with human skin, clinically acceptable, stable, and cost-effective, allowing it to play a significant role in monitoring daily health and wellness, as well as in the prevention, diagnosis, treatment, and prognosis of diseases. Additionally, this technology has the potential to be particularly impactful in sports activities, where continuous monitoring is essential for optimizing performance and preventing injuries.

## Figures and Tables

**Table 1:** Performance comparison of sweat collection and detection using different innovative materials. CA: Chronoamperometry. CV: Cyclic Voltammetry. CP: Chronopotentiometry. DPV: Differential Pulse Voltammetry. ECL: electrochemiluminescence. EIS: Electrochemical Impedance Spectroscopy. MIPs: molecularly imprinted polymers. OCP: Open-Circuit Potential. SWV: Square Wave Voltammetry. LOD: limit of detection.

	Material Type	Method	Analyte	Detection Range	Sensitivity	Ref.
Innovative materials for sweat collection	PDMS and nanofiber films	MIP	cortisol	1 nM–1 μM		[70]
	PDMS	CA	uric acid	2–250 µM, LOD: 1.2 μM	0.875 µA/µM/cm^2^	[64]
	PDMS	EIS	sweat rate and total electrolyte concentration	Sweat rate: 0.5–20 μL/min/cm^2^, Total electrolyte concentration: 1–200 mM		[65]
	PDMS	CA	uric acid	2–70 μM, LOD: 0.34 μM		[66]
	PDMS	EIS	cortisol	0.01–100 nM, LOD:1 pg/mL		[67]
	PDMS	DPV	cortisol	1 pM to 1 μM, LOD 0.2 pM		[68]
	PDMS	CA	lactate	0–10 mM	36.2 μA/mM/cm^2^	[69]
	PDMS	CA	glucose and lactate			[53]
	hydrogel	CA	glucose	6.25 μM to 0.8 mM		[62]
	hydrogel	CA, EIS	glucose and cortisol	Glucose: 5.0–50 μM, Cortisol: 1.0–15 ng/mL		[63]
	paper	MIP	cortisol	1 nM–1 μM		[55]
	paper	CA	glucose and lactate	Glucose: 0.08–1.25 mM, Lactate: 0.3–20.3 mM	Glucose: 2.4 nA/μM, Lactate: 0.49 μA/mM	[52]
	cloth	CA	glucose	0.05–1 mM	105.93 μA/mM/cm^2^	[60]
	Silicone and PET	EIS	sweat rate and chloride			[71]
	PET	EIS	sweat rate, conductivity, and copper levels	0–2500 ng/mL, LOD: 396 ng/mL	2.3 nA/ng/mL	[51]
	PET	CA	glucose	0.1–1 mM, LOD: 24 μM	1.27 μA/mM/cm^2^	[72]
	fabric with Janus structure	CA, CP	glucose, Na^+^, K^+^, NH_4_^+^, and pH levels	Glucose: 0–500 µM, NaCl: 10–160 mM, KCl: 2–32 mM, NH_4_Cl: 10–160 mM, pH: 4–7	Glucose: 3.53 nA/µM, Na^+^: 48.4 mV/decade, K^+^: 51.2 mV/decade, NH4^+^: 53.9 mV/decade, pH: 47.8 mV/decade	[61]
	fabric with Janus structure	OCP, CA	Na^+^, pH, and glucose	Na^+^: 5–160 mM, pH: 4 to 8	Na^+^: 53.83 mV/decade, pH: 98.68 mV/decade, glucose: 0.04 μA/μM	[56]
	cotton	CP	Li^+^, Pb^2+^, K^+^ and Na^+^	LOD of Li^+^: 1.4 ± 0.2 mM, LOD of Pb^2+^: 4.13 ± 0.23 mM, LOD of K^+^: 3.10 ± 0.10 mM, LOD of Na^+^: 14.30 ± 5.13 µM	Li^+^: 57.6 ± 2.1 mV/decade, Pb^2+^: 58.8 ± 1.4 mV/decade, K^+^: 55.1 ± 0.9 mV/decade, Na^+^: 28.9 ± 1.64 mV/decade	[59]
Innovative materials for sweat detection	hydrophobic paper	EIS, CP, OCP	K^+^, Na^+^, Cl^−^, and pH			[75]
	fiber/fabric	SWV	IL-6	1 pg/mL to 100 ng/mL, LOD: 280 fg/mL		[76]
	fiber/fabric	CV	glucose	0.1–0.6 mM, LOD: 301 ± 2 nM	126 ± 14 nA/mM	[77]
	PVC, PI, and PET	CP	K^+^, Na^+^, and Ca^2+^	LOD of K^+^: 10 mM, LOD of Na^+^: 10 mM, LOD of Ca^2+^: 100 mM		[78]
	PI	CV, DPV	uric acid (UA), tyrosine (Tyr), and ascorbic acid (AA)	UA, AA and Tyr: 10–160 μM. LODs of UA: 0.47 μM, LOD of AA: 1.25 μM, LOD of Tyr: 14.38 μM		[79]
	CNT-EVA film	CV, CA	glucose	LOD: 3 μM	270 ± 10 μA/mM/cm^2^	[34]
	PDMS and lignin-LIG	CP	Na^+^ and K^+^	Na^+^: 0.1 μM to 0.1 M, K^+^: 0.01 μM to 0.1 M	Na^+^: 63.6 mV/decade, K^+^: 59.2 mV/decade	[80]
	multilayer PDMS	MIPs	pH, epinephrine, dopamine, and lactate	pH: 4.25–10, LOD of epinephrine: 0.7 ± 0.2 nM, LOD of dopamine: 2.11 ± 0.05 nM, LOD of lactate: 0.07 ± 0.07 mM		[81]
	conductive hydrogel	OCP, DPV, CV	pH and tyrosine (Tyr)	Tyr: 10–200 μM (R^2^ = 0.9985), LOD: 3.3 μM	pH: −71.86 mV/pH	[82]
	hydrogel and TPU	OCP	pH, Na^+^, K^+^	pH: 4–9, Na^+^: 5–160 mM, K^+^: 1–32 mM	pH: 58.14 mV/pH, Na^+^: 58.89 mV/decade, K^+^: 59.11 mV/decade	[83]
	rGO@ hydrogel and Lum@ hydrogel	ECL	urea, lactic acid, and chlorion			[35]

**Table 2:** Performance comparison of self-powering sweat sensors using different innovative materials. Output voltage: voltage provided by generators to the external load. Open-circuit voltage: voltage when the circuits are not connected to the load.

Material Type	Method	Generator Size	Voltage (V)	Analyte	Power Density	Ref.
PVDF/graphene and poly (3-aminophenyl boronic acid) (3-APBA)	triboelectric nanogenerator (TENG)		Output voltage of 30 V	Lactate		[36]
MWCNTs/polyaniline (PANI)/Ecoflex fiber, and twining varnished wires	fiber-based triboelectric nanogenerators (F-TENGs)	2 mm (diameter)	Open-circuit voltage of 3 V	Glucose, creatinine and lactate acid		[86]
Teflon (PTFE)–ZnO	Single-electrode triboelectric nanogenerator (STENG)	10 mm (diameter)	Open-circuit voltage of 5 V	Cl ions	2.5 µW/cm^2^	[87]
PET, Ag, and PVDF	piezoelectric nanogenerators (PENGs)	40 mm × 15 mm	Output voltage of 97 V	Na^+^, K^+^, and pH	140 mW/m^2^	[88]
Carbon film@LOx	water-evaporation nanogenerator	25 mm × 25 mm	Output voltage of 0.2 V	Lactate		[45]
NQ/MWCNT; PB/MWCNT	enzymatic biofuel cell (BFC)	35 mm × 15 mm	Open-circuit voltage of 0.45 V	Glucose	266 μW/cm^2^	[48]
3D-NHCAs, alcohol oxidase (AOx), bilirubin oxidase (BOx), and TPA	ethanol biofuel cell	40 mm × 45 mm		Ethanol	1.9 μW/cm^2^	[89]
SWCNTs@Cu; zinc foil	redox reactions	50 mm (diameter)	Output voltage of 0.8 V	EMG	18.3 μw/cm^2^	[90]
Zn-wire core, cotton yarn, and carbon yarn	redox reactions	1.6 mm (diameter)	Output voltage of 0.73 V	The arm swing frequency and breathing rate	1.72 mW/cm^2^	[91]
Cotton, Mg, cotton/KCl, Ni/G Foam	redox reactions	40 mm × 20 mm	Open-circuit voltage of 1.4 V	temperature, pulse rate (PR), and oxygen saturation in blood (SpO_2_)	16.3 mW/cm^2^	[92]
